# Hierarchical Cluster Analysis of Medical Chemicals Detected by a Bacteriophage-Based Colorimetric Sensor Array

**DOI:** 10.3390/nano10010121

**Published:** 2020-01-09

**Authors:** Chuntae Kim, Hansong Lee, Vasanthan Devaraj, Won-Geun Kim, Yujin Lee, Yeji Kim, Na-Na Jeong, Eun Jung Choi, Sang Hong Baek, Dong-Wook Han, Hokeun Sun, Jin-Woo Oh

**Affiliations:** 1 Department of Nanofusion Technology, Pusan National University, Busan 46241, Korea; chuntae1122@gmail.com (C.K.); kim1guen@gmail.com (W.-G.K.); pinky204@hanmail.net (Y.L.); kkyeaj0153@naver.com (Y.K.); 2 Interdisciplinary Program of Genomic Data Science, Pusan National University, Busan 46241, Korea; hansong798@naver.com; 3 Department of Statistics, Pusan National University, Busan 46241, Korea; 4 Research Center for Energy Convergence and Technology, Pusan National University, Busan 46241, Korea; vasanth@pusan.ac.kr (V.D.); eunjung721203@gmail.com (E.J.C.); 5 BK21PLUS Program in Embodiment: Health-Society Interaction, Department of Public Health Sciences, Graduate School, Korea University, Seoul 02841, Korea; nana8931@naver.com; 6 Laboratory of Cardiovascular Disease, Division of Cardiology, School of Medicine, The Catholic University of Korea, Seoul 06591, Korea; whitesh@catholic.ac.kr; 7 Department of Cogno-Mechatronics Engineering, College of Nanoscience & Nanotechnology, Pusan National University, Busan 46241, Korea; 8 Department of Nanoenergy Engineering, Pusan National University, Busan 46241, Korea

**Keywords:** M13 bacteriophage, multi-array sensors, hierarchical cluster analysis, high selectivity

## Abstract

M13 bacteriophage-based colorimetric sensors, especially multi-array sensors, have been successfully demonstrated to be a powerful platform for detecting extremely small amounts of target molecules. Colorimetric sensors can be fabricated easily using self-assembly of genetically engineered M13 bacteriophage which incorporates peptide libraries on its surface. However, the ability to discriminate many types of target molecules is still required. In this work, we introduce a statistical method to efficiently analyze a huge amount of numerical results in order to classify various types of target molecules. To enhance the selectivity of M13 bacteriophage-based colorimetric sensors, a multi-array sensor system can be an appropriate platform. On this basis, a pattern-recognizing multi-array biosensor platform was fabricated by integrating three types of sensors in which genetically engineered M13 bacteriophages (wild-, RGD-, and EEEE-type) were utilized as a primary building block. This sensor system was used to analyze a pattern of color change caused by a reaction between the sensor array and external substances, followed by separating the specific target substances by means of hierarchical cluster analysis. The biosensor platform could detect drug contaminants such as hormone drugs (estrogen) and antibiotics. We expect that the proposed biosensor system could be used for the development of a first-analysis kit, which would be inexpensive and easy to supply and could be applied in monitoring the environment and health care.

## 1. Introduction

**Figure 1:**
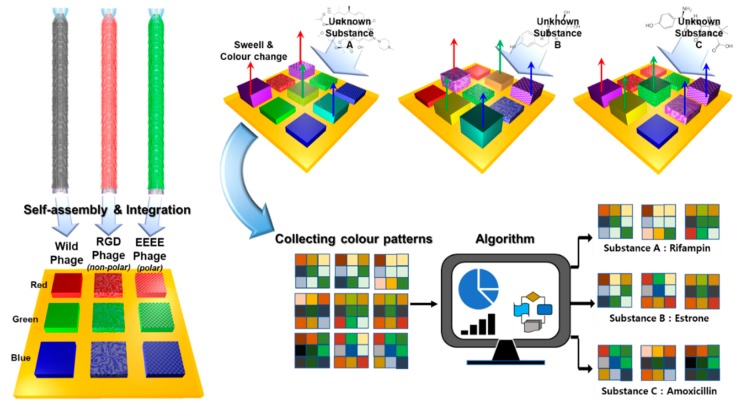
Schematic illustration of the M13 bacteriophage-based colorimetric sensor array. The sensor array consists of a functional M13 bacteriophage with a modified major coat protein (pVIII). When a color band is reacting with a target analyte, each type of sensor chip shows its own color change value according to the individual M13 bacteriophage’s characteristics. A color pattern is formed as a unique response value, and it is possible to construct a sensor platform which can discriminate unknown textures through pattern analysis.

M13 bacteriophage, one kind of filamentous bacteriophages, have been utilized as receptors in biosensors [1,2]. By means of genetic engineering techniques, it is possible to modify and achieve a better binding affinity of M13 bacteriophage towards desired target molecules [3,4,5]. Among various types of M13 bacteriophage-based biosensors, colorimetric sensor systems have been intensively investigated due to their facile fabrication process and sensing method [6,7,8]. Colorimetric sensors fabricated by self-assembly of M13 bacteriophage result in nanostructures with varying size and periodicity [9]. When white light is illuminated onto the nanostructure, specific wavelengths determined by Bragg’s law are scattered more dominantly from the nanostructures. After the penetration of external chemicals, the self-assembled nanostructures swell, resulting in a change of the wavelengths that are scattered [10]. The observed color change is detected by a complementary metal–oxide–semiconductor detector, and this is followed by image analysis. The image analysis results consist of numerical values that can be used to determine the type and concentration of the external chemicals. Using genetic engineering techniques, M13 bacteriophage-based colorimetric sensors can display sensitive color changes toward desired target materials. Furthermore, by integrating various types of genetically engineered M13 bacteriophage-based colorimetric sensors on a single chip to fabricate a sensor array, a number of target molecules can be classified by analyzing the pattern of a color change. Even though M13 bacteriophage-based multi-array sensor systems have been successfully demonstrated as a powerful platform for the detection of extremely small amounts of target molecules, the discrimination of many types of target molecules is still both necessary and challenging. However, as the types of genetically engineered M13 bacteriophage integrated on sensor arrays increase, the analysis of the sensing results becomes complicated due to the huge amount of numerical data. In this work, we introduce a statistical method, henceforth called hierarchical cluster analysis, to classify many types of medical chemicals. Antibiotics are used widely in the livestock industry on a daily basis, but the excessive use of antibiotics raises the problem of increased antibiotic resistance in animals and humans [11]. If estrogen compounds, such as estrone (E1), 17β-estradiol (E2), and estriol (E3), and oral contraceptives released from humans and animals such as endocrine disrupters (similar to 17α-ethinylestradiol (EE2)) contaminate the environment through sewage and the excrement of animals, they can adversely affect the ecology in the water [12]. Estrogenic and antibiotic substances are found in trace amounts (~ng/L) in effluent, fresh water, river water, and even in drinking water [13,14], as a consequence of their ineffective removal at wastewater treatment plants (WWTP). In general, as any particular substance is not always present by itself, an effective method to comprehensively classify unknown compounds is necessary [15]. In this regard, M13 bacteriophage-based sensor arrays and hierarchical cluster analysis are a suitable sensor system to detect and classify various types of unknown compounds. Figure 1 is a schematic illustration describing our sensor system.

## 2. Materials and Methods

### 2.1. Sensor Analysis Analytes

Four types of estrogen drugs and four types of antibiotics were purchased from Sigma-Aldrich (Seoul, South Korea) and a local pharmacy (Medipharm, Busan, South Korea), ground, and used directly. In real-world situations, all such analytes exist in various physical conditions that cause molecules to vibrate or diffuse. To mimic these kinds of physical conditions, sensing experiments were carried out at four different temperatures (30, 60, 90, and 120 °C) for each material.

### 2.2. Development of Functional Bioreporter Materials

The wild types of filamentous phage particles were based on M13KE, which was purchased from New England Biolabs (Ipswich, MA, USA). The major coat protein (pVIII) of an M13 bacteriophage with an arginine-, glycine-, and aspartic acid-based functional peptide sequence (-RGD-) [16] and a glutamic acid-based peptide sequence (-EEEE-) were fabricated on M13KE phage [17].

### 2.3. Phage Colorimetric Sensor-Based Multi-Array Chip

A silicon wafer with a 100 nm gold film on a 5 nm platinum adhesion layer was selected as a substrate to deposit the M13 bacteriophage. Simple pulling methods [18] were utilized to prepare three kinds of colorimetric sensors consisting of wild-, RGD-, and EEEE-type M13 bacteriophages. The three types of colorimetric sensors consisted of red-, green-, and blue-colored M13 bacteriophage films that were determined by pulling speeds of 30 μm/min, 40 μm/min, and 50 μm/min, respectively. The fabricated colorimetric sensors were integrated into a single chip.

### 2.4. Colorimetric Signal Analysis and Data Processing

The color change was observed using a handheld digital microscope (Celestron DELUXE HANDHELD DIGITAL MICROSCOPE, Torrance, CA, USA) in the chamber of a color sensor array set made from phage chips. The colorimetric signal of the unit cell constituting each sensor chip was measured as the average value of a ~1000 pixel square. A method of setting the values of ΔR, ΔG, and ΔB was used, and the changes in each variable were used as the criteria for the amount of color change [7].

### 2.5. Analytical Data Statistics

Essentially, model-less clustering analyses, such as principal component analysis or hierarchical clustering analysis, use hierarchical analysis because they are unsuitable for predictive (i.e., classificatory) use. Hierarchical clustering analysis is a statistical analysis that classifies nearby objects into the same group using the distance that indicates the similarity of each object [19]. The method is called hierarchical cluster analysis because it forms a tree-like hierarchical structure by starting from objects at the closest distance and combining them. Generally, Euclidean distances are commonly used for distance calculations. On the other hand, the color distance * is used so that the color characteristics of RGB can be utilized, as the data used for the analysis are RGB data, representing a color change value that occurs when one sample reacts with another sample.

$$\overset{n}{\underset{i=1}{\sum }}\sqrt{\left({\left(R_{i}^{a}-R_{i}^{b}\right)}^{2}+{\left(B_{i}^{a}-B_{i}^{b}\right)}^{2}+{\left(G_{i}^{a}-G_{i}^{b}\right)}^{2}\right)}$$

* Color distance: The color distance metric calculates the Euclidean distance in color space between each pair of clusters, ignoring their size. The distance between images a and b (using RGB) was calculated as in the above expression, where n is the number of bins.

## 3. Results and Discussion

**Figure 2:**
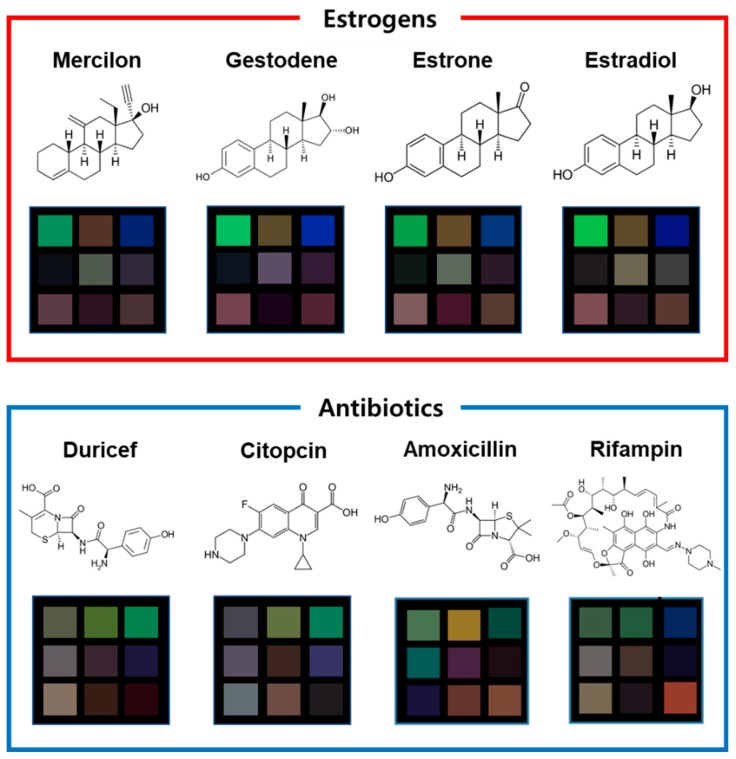
Image of the colorimetric sensor array chip after exposure to medical chemicals. The color pixels represent the mean value of the variation of the RGB values (in 8 bit) as a function of the M13 bacteriophage bundle’s structural change.

**Figure 3:**
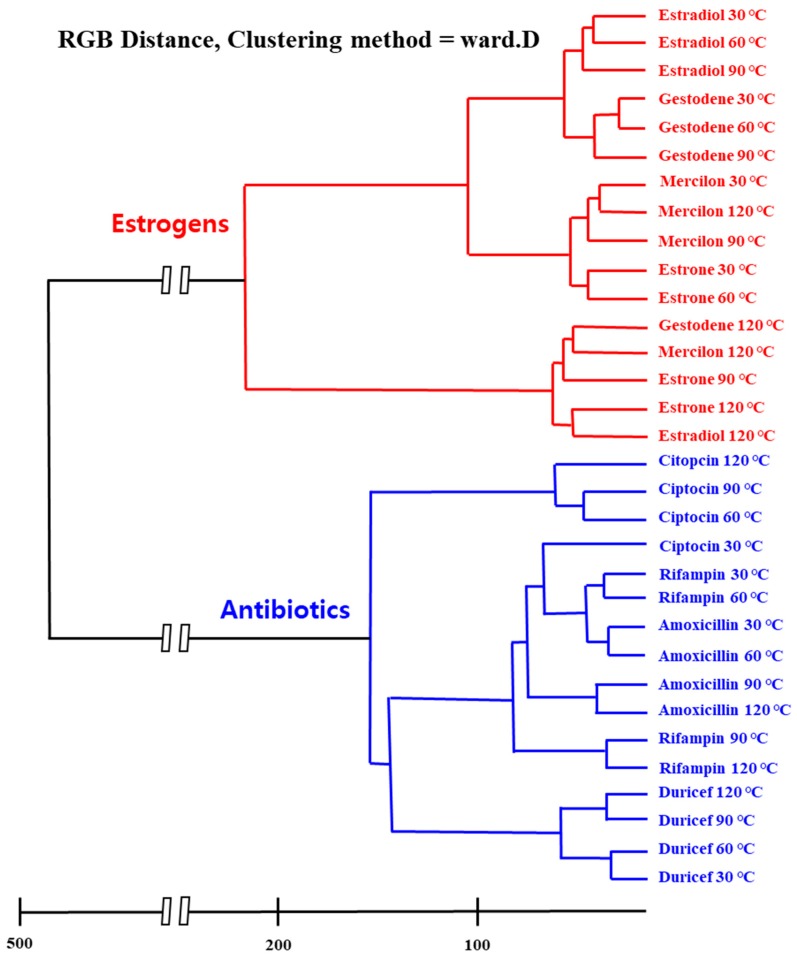
Hierarchical cluster analysis dendrogram for eight types of medical chemicals using the Ward.D linkage method, which is based on the linear model criterion of least squares. The Euclidean distance was set to the ΔRGB matrix which is made of the colorimetric shift values.

Figure 1 depicts a schematic illustration of the M13 bacteriophage-based multi-array sensor chip and color-pattern analysis process. The multi-array sensor chip shows specific color patterns according to the applied materials due to the different chemical groups expressed on the M13 bacteriophage. Furthermore, microstructures of colorimetric sensors determined by the pulling speed contribute to the color change, owing to their different surface-to-volume ratios [7,8,10,18]. After collecting the color patterns, a statistical analysis process determines the types of unknown substances. Figure 2 displays the specific color patterns toward each target chemical at a temperature of 120 °C. The specific color patterns were generated by calculating pixel differentiation between initial color and color after exposure to the target chemical. As shown in Figure 2, the M13 bacteriophage-based sensor array displays specific color change according to the exposed chemicals. However, because this was a qualitative analysis, there were limitations in the exact classification of the target chemicals. The M13 bacteriophage-based sensor array, different from specialized sensors such as litmus paper or a pregnancy tester, is a non-specific sensor system, which means small amounts of color changes should be analyzed and classified exactly. Signal detection and data analysis by the sensor of the drug component are based on the inherent chemical properties of the material. The analysis of female hormone-related drugs, antibiotic components, and their clustering tendencies were analyzed through various features. Through the RGB distance values, the Euclidean distance was set to the RGB distance, and a hierarchical classification was performed using the Ward Linkage (Ward.D) method [20] (see Figure 3). The similarity between two clusters was measured on the basis of the increment of the error sum of squares (ESS) when two clusters were combined. The increase of ESS was measured as the distance between two clusters when the distance matrix was obtained. This method was less sensitive to noise or outlier data than the Single Linkage Method [20]. The clustering results showed a close relationship with each chemical composition. They confirmed that estrogen-based drugs and antibiotics-based drugs can be classified within a large frame and partially. In particular, the similar chemical structures of antibiotic substances made their distances very close to each other. Their vapor pressure values are similar to each-other at various temperatures, and these compounds react comparably with sensor array chips. The similar composition of the pharmaceutical forms containing each compound also had an impact.

## 4. Conclusions

We fabricated a functional M13 bacteriophage-based photonic crystal structure and set a complex detection sensor device with three kinds of sensor chips with chemically inherent functional groups. We propose a sensor model that can classify antibiotics and hormone drugs by analyzing the color change amount of a 3 × 3 squared band, i.e., nine sections by setting three color bands per chip. In particular, commercial chemicals can be classified according to the product brand, so that the clustering results can slightly overlap. We see the possibility of integrating this method in a simple sensor kit for quality assurance and quality control of commercial drugs. This colorimetric sensor array model could be used to fabricate a very simple sensor platform consisting of a sensor chip 1 square centimeter wide, a chamber of about 30 cc capacity, and a small webcam for color change observation. The multi-array sensor system proposed in this work consists of nine different types of sensor chips, but in the future, by increasing the types of sensors, the selectivity of the multi-array sensor system could significantly ameliorate. Furthermore, our multi-array sensor platform could be used for user-friendly pre-analyses that could be simple and performed in real time, before instrument-based chromatographic analyses.
